# Exploring the rewards and challenges of paediatric palliative care work – a qualitative study of a multi-disciplinary children’s hospice care team

**DOI:** 10.1186/s12904-017-0254-4

**Published:** 2017-12-16

**Authors:** Johanna Taylor, Jan Aldridge

**Affiliations:** 1 Department of Health Sciences, University of York, Heslington, York, YO10 5DD UK; 2 Martin House Hospice Care for Children and Young People, Grove Road, Boston Spa, West Yorkshire LS23 6TX UK

**Keywords:** Paediatric palliative care, End of life, Children’s hospice, Work-related stress, Burnout, Staff support, Qualitative research, Clinical reflection, Clinical supervision

## Abstract

**Background:**

Children’s hospices are a key provider of palliative care for children and young people with life-limiting and life-threatening conditions. However, despite recent policy attention to the provision of paediatric palliative care, little is known about the role of children’s hospice staff and the factors that may impact on their wellbeing at work. This study explored the rewards and challenges of working in a children’s hospice with an aim to identify staff support and development needs.

**Methods:**

We conducted an exploratory, qualitative study involving thematic analysis of semi-structured interviews with 34 staff and three focus groups with 17 staff working in a multi-disciplinary care team in a UK children’s hospice.

**Results:**

Participants identified rewards and challenges related to the direct work of caring for children and their families; team dynamics and organisational structures; and individual resilience and job motivation. Participants described the work as emotionally intensive and multi-faceted; ‘getting it right’ for children was identified as a strong motivator and reward, but also a potential stressor as staff strived to maintain high standards of personalised and emotional care. Other factors were identified as both a reward and stressor, including team functioning, the allocation of work, meeting parent expectations, and the hospice environment. Many participants identified training needs for different aspects of the role to help them feel more confident and competent. Participants also expressed concerns about work-related stress, both for themselves and for colleagues, but felt unable to discuss this at work. Informal support from colleagues and group clinical reflection were identified as primary resources to reflect on and learn from work and for emotional support. However, opportunities for this were limited.

**Conclusions:**

Providing regular, structured, and dedicated clinical reflection provides a mechanism through which children’s hospice staff can come together for support and learning, and demonstrates an organisational commitment to staff wellbeing and development. Being aware of children’s hospice specific rewards and challenges can help to ensure that staff feel supported and competent in their role. Breaking down barriers to discussing work-related stress and enhancing awareness about early signs of burnout is also important.

## Background

Children’s hospices are identified as a key provider of paediatric palliative care, which is defined as “the active total care of the child’s body, mind and spirit” [1] and supports children and young people with life-limiting and life-threatening conditions from diagnosis through end of life and their families in bereavement [2, 3]. In the UK, children’s hospices support more than 7000 children and young people each year [4], which represents around 15% of those diagnosed with a life-limiting condition [5]. A similar proportion of children and young people in the US receive hospice care, although due to the smaller number of children’s hospices in the US, this is predominantly provided by children’s hospitals and adult hospices [6].

Paediatric palliative care is now recognised as a distinct specialty which involves multi-disciplinary teams working across numerous organisations including hospitals, community settings, and children’s hospices [2, 3]. The important role of the staff working in this specialty has been acknowledged in numerous reviews and guidelines internationally [7–10], and educational standards and staff competencies are now being developed [11]. However, relatively little consideration has been paid to the specific challenges of the work, [11] and studies highlight the limited organisational and emotional support available for staff [12, 13].

Paediatric palliative care has distinct differences from adult palliative care which generally supports the end of life phase of care [14], and from paediatric oncology which although similarly supports children and their families sometimes for many years including during end of life [15], has a pathway that is focused primarily on remission and cure [16]. In paediatric palliative care the key focus is on palliation, including medical, physical, emotional, spiritual and social care, as well as providing continuous care at the end of life and help in bereavement [17]. For staff this can mean re-negotiating traditional professional boundaries to provide individualised care that aims to enhance quality of life for children and their families [18].

Children’s positioning within the family system also has implications for the work, with the family being the unit of care [19]. For children with life-limiting conditions, parents are often experts in their child’s care and assume responsibility for carrying out a range of health-care procedures [20]. As well as working in partnership with parents and supporting siblings, the staff must respect the evolving role of children in decision-making about their treatment and care [21]. This can involve assessing the benefits and drawbacks of complex medical interventions and life-sustaining technologies, and anticipating the clinical implications of these treatments on the developing child [22].

A recent systematic review exploring staff’s experience in providing end-of-life care across a range of settings found that the work is both rewarding and challenging [13]. The review identified the close relationships with families and the importance of the work of supporting children as key rewards. Maintaining appropriate boundaries and experiencing grief over the loss of a child were identified as key challenges associated with this; other challenges included time constraints caused by demanding caseloads and staff shortages; communication difficulties with parents, colleagues and management; and barriers to co-ordinating care with other agencies. End-of-life decision making and symptom management were the most commonly cited challenges, although the review observed that those with expertise or experience in palliative care were more comfortable in providing end-of-life care.

The review described the children’s hospice setting as the most optimal work environment when compared to hospital and community settings, referring to the informal and home-like setting, and the priority and time afforded to social, emotional and spiritual aspects of care [13]. However, only two of the 16 studies focused on children’s hospice work [18, 23] even though they are a primary provider of palliative care [7], and the review authors highlight the paucity of evidence exploring staff experiences in this setting.

This paper aims to address this gap, reporting the findings of a qualitative study exploring work-related challenges and rewards, and support and development needs in a multi-disciplinary care team in one children’s hospice.

## Methods

A qualitative exploratory design was used, adopting an interpretive lens that focuses on the multiple-perspective stories of individuals working in a children’s hospice [24]. Methods included thematic analysis of data from semi-structured interviews and focus groups with care staff.

### Setting

The multi-disciplinary care team of a large and well established children’s hospice in England, providing respite and end-of-life care for children and young adults and their families.

### Study participants

To explore multiple perspectives on the children’s hospice role, all care team members not on maternity leave or long-term absence were invited to take part in an interview. A purposive sampling strategy was employed during recruitment to ensure individuals with different roles, backgrounds and time in post took part. In total, 34 care team members agreed to take part and participated in an interview (see Table 1 for interview participant roles).

**Table 1:** Interview participant roles

Job role	Number of participants
Medical and nursing staff (doctors, registered general and specialist nurses)	10
Other care team staff (allied health professionals, nursery nurses, therapists, and care staff with other qualifications and skills)	20
Managers and other hospice staff	4
Total participants	34

Focus groups were then used to further explore work-related rewards and challenges distinct to the children’s hospice environment, using the shared identity and experience of the group to generate discussions that may not have occurred during individual interviews [25, 26]. A convenience sampling strategy ensured groups could be convened, although efforts were made to include staff with different professional backgrounds in each group to encourage debate and diverse viewpoints (staff cover was arranged to facilitate attendance). Three focus groups were planned, each containing around six participants; this would allow for individual contribution as well as group discussion, and enable differences between groups to be explored [26]. In total, 17 care team members took part (three participants also took part in an interview), with group sizes ranging from four to seven (see Table 2 for focus group participant roles).

**Table 2:** Focus group participant roles

Job role	Number of participants
Medical and nursing staff	10
Other care team staff	7
Managers and other hospice staff	0
Total participants	17

As well as varying in occupational background and professional training, the 48 participants had worked at the hospice for different durations, ranging from 10 months to more than 20 years. Eight males and 40 females took part in the study. A total of 10 participants had experience of supporting children and young people with life-limiting or life-threatening conditions prior to working at the hospice. Only a few participants had received any training in paediatric palliative care (exact number not provided to protect the anonymity of participants).

### Data collection

An interview topic guide was used to explore the rewards and challenges of work and staff support and development needs. The topic guide was based on materials developed for stage one of a study conducted by Mukherjee et al. [27], which interviewed staff to identify work-related demands and rewards specific to paediatric oncology. In Mukherjee et al.’s study, the definitions in Table 3 were presented to interviewees, and used as a basis to explore sources of stress, reward and satisfaction. As well as successfully capturing the range of demands and rewards encountered by participants, this approach yielded rich qualitative data on the experience of working in paediatric oncology, including supporting children and young people during the end of life phase. The same approach was therefore used in the study reported here, with topic guides adapted to reflect the children’s hospice setting and palliative care focus.

**Table 3:** Definition of work-related demands and rewards

Work-related ‘DEMANDS’	Work-related ‘REWARDS’
‘Things which you have found, or are finding, difficult, upsetting, annoying, challenging and / or a hassle’ *Demands can be:* *One-off / Occasional / Regular / Everyday* *Things that are demanding at certain times and not others*	‘Events or situations which you experience, or things which you do or have done, which have been, or are, rewarding’ *Rewards can be:* *One-off / Occasional / Regular / Everyday*

Adapted from Mukherjee et al. [27]

All interviews were conducted face-to-face by JT, either in a private room at the hospice or in participants’ homes. Interviews were audio recorded and transcribed (intelligent verbatim) with participants’ consent and destroyed after data analysis. They ranged from 33 to 76 min (median duration 47 min).

The focus groups were run by JT, with JA assisting in facilitation and note taking. An hour was allocated for each focus group due to staff cover arrangements. First, in small groups, participants discussed and wrote down the demands and rewards associated with their work, again using the definitions in Table 3. The whole group then discussed these together to explore similarities and differences between participants, and understand more about why certain factors relating to work were identified as demands or rewards. These lists and detailed field notes taken by JT and JA (during and following each focus group) were used as data; we did not audio record the focus groups due to concerns about participant confidentiality and anonymity.

All data were collected between December 2010 and May 2011.

### Data analysis

Interview and focus group data were analysed thematically to explore work-related challenges and rewards and to examine other themes within the data that pertained to work-related stress or staff support and development. Field notes and participant lists from each focus group formed a unit of analysis, and were analysed alongside interview transcripts rather than conducting separate analyses. This approach was adopted in part due to concerns about anonymity (see section on Ethical considerations) but also because we were interested in exploring the range of rewards and challenges experienced by children’s hospice staff rather than the differences expressed by individuals versus groups, and each data source contributed to this understanding.

Using principles of the Framework method, data analysis involved a process of familiarisation (through reading and re-reading of data); identification and organisation of challenges, rewards, support and development needs, and other themes in the data (through making notes on transcripts and focus group data, highlighting key words and concepts, listing and grouping themes together to create a coding framework); coding of data segments (defined as extracts of meaning relating to themes and subthemes); and comparison across and within cases to explore further the patterns of themes identified and develop higher order explanatory themes [28].

Three researchers analysed and interpreted the data (JT, BW, JA). On-going reflection between JT and JA to refine themes and identify relationships between themes in the data formed part of the analytic process.

### Research quality

Study quality was assured against several criteria, including dependability, credibility and authenticity [29]. The use of topic guides for interviews and focus groups provided a standardized approach to data collection and a detailed audit of processes was kept to create dependability. A good sampling strategy, including assessment of data saturation during data collection to determine the final sample size for interviews, helped assure credibility. To assure authenticity, two meetings were held with care team members following the study to discuss emerging findings; this led to good respondent validation and feedback. The findings have also been discussed with other hospices and presented at national conferences; feedback confirms that the key findings resonate with the experience in other settings.

### Ethical considerations

For this study, specific consideration was given to the process of informed consent, confidentiality and anonymity, the presentation of findings, and participant distress.

#### Informed consent

The children’s hospice involved gave overall permission for the research team to conduct the study, and in doing so, access to its staff. Therefore, to ensure that staff did not feel pressured to take part they were invited in writing by the research team rather than the organisation, and provided with comprehensive information about the study, why it was being carried out, and how their information would be used.

#### Confidentiality and anonymity

Members of staff who were interested in taking part in an interview were asked to contact the research team directly either by phone or email, or by completing a response form and returning it to the researcher. This was to ensure their participation remained confidential. As this was not possible for focus groups because of their timing and location, the decision was taken to use field notes rather than audio recordings. This also informed the decision to combine interview and focus group data during analysis.

Care was taken to ensure that participants could not be identified in presentations or reports, particularly as it was likely that the children’s hospice was identifiable. While it was important that participant characteristics were provided to aid the reader, this was censored by the need to ensure that the anonymity of participants was maintained.

#### Presentation of findings

Participants and others involved in providing feedback on the study were keen that we present our findings in full to show the overlapping and sometimes conflicting nature of work-related challenges and rewards, and the negative as well as positive aspects of working in a children’s hospice. This informed our decision to provide sufficient detail when reporting the findings and to include reference to the data for each main analytical theme.

#### Participant distress

Because of the study topic and exploratory design, it was possible that some participants might become distressed during interview, or identify themselves as being at risk of, or experiencing, work-related stress or burnout. To ensure that participants were able to follow this up should they wish to, they were directed to the organisation’s clinical psychologist.

## Results

Seven main themes identified work-related challenges and rewards; *making a difference and getting it right; a multi-faceted role; complexity of children’s care; team functioning; being valued; individual coping; and job motivation*. These themes fed into three over-arching categories impacting on wellbeing at work: doing palliative care work; team and organisational dynamics; and individual resilience (see Table 4).

**Table 4:** Themes identifying work-related challenges and rewards

Overarching categories	Main analytical themes
Doing palliative care work	Making a difference and getting it right A multi-faceted role Complexity of children’s care
Team and organisational dynamics	Team functioning Being valued
Individual resilience	Individual coping Job motivation

### Doing palliative care work

Participants portrayed a ‘*multi-faceted role’* that required a combination of different skills, traits and knowledge. It was a generic role across the team, and participants explained that all care team members were expected to meet the holistic needs (physical, emotional, social and spiritual) of the children and families they supported.

Many participants talked about ‘*making a difference*’ in their role to help enhance families’ quality of life, being aware of the challenges families face in everyday life. This insight motivated participants to provide high standards of holistic care, beyond meeting physical and medical needs. It was important and rewarding to participants that they were ‘*getting it right*’ and that the families received a good service.

Many participants explained that an emotional investment was necessary to achieve this, which they believed could place them at risk of distress as they formed close relationships with children and their families.

*“This job is physical, mental and psychological. You’re giving [your] whole. It’s like no other job I’ve ever done.”* (participant 31)

Some participants explained that the commitment to get it right and the informal relationships formed with families could make it difficult to say no to requests from families (e.g. trips out or activities for siblings). This was reported to place additional pressures on the team, especially during busy times or when staff numbers were low.

*“I think sometimes we try to please too many people most of the time and then we have shifts where it all goes wrong … you might have ten families and really we have barely enough staff to give them the kind of care that we are promoting.”* (participant 26)

Some participants explained that they were reluctant to ask for help during these times, and described the efforts to maintain a positive front for families and to put themselves under additional pressure to ‘get it right’.

*“There are times when there are some very poorly kids and if there is only (a few) of you on [shift] it is very physical and it is very demanding … but I think sometimes we just carry on coping and it is like, we aren’t very good at asking for help.”* (participant 35)

Many participants described feelings of distress or disappointment when they were not able to meet families’ needs. Having an opportunity to debrief (formally or informally) after particularly challenging events or shifts played an important supportive function, although many participants explained that these opportunities were limited. Participants identified group clinical reflection (facilitated groups in which colleagues have an opportunity to explore and discuss their experiences and reflect critically and systematically on particular issues or matters raised by the work) as useful to reflect on difficult or distressing aspects of the work as a team.

*“It’s just looking at things in a bit more depth and interesting to hear different people’s attitudes… making the understanding a bit more clear, or giving you a different perspective on something, or feeling that if you’re in that situation again you might be able to deal with it a bit more competently.”* (participant 5)

All team members performed the cares that parents provided at home. The *‘complexity of children’s care’* varied, with some children requiring very little clinical care and others requiring very complex and individualised care. Some participants noted that the time required for this aspect of the work could impact negatively on the wider role of meeting social and emotional needs.

*“It is like intensive care sometimes … and there is all this new equipment and there is like more and more expected of us … we are not spending quality time with them [children].”* (participant 35)

Several participants who had worked in a children’s hospice for a number of years believed that the proportion of children with complex care regimes and life-sustaining technologies had increased, reflecting advances in medical technology. This, they explained, had increased needs for training and support.

*“The world has changed; the equipment for the children has changed and we have to change with that.”* (participant 4)

The skills required to carry out certain nursing tasks and manage the range of life sustaining technologies was reported to place both nursing and other staff outside their comfort zone.

*“One minute you are having to deal with let’s say … TPN [total parenteral nutrition], and we have had quite a lot of input about that … then the next minute you have got the child on a ventilator and then another time you have got tracheotomies … that is where the anxiety comes.”* (participant 17)

Looking after a child whose care was unfamiliar was identified as a key stressor. Some participants described a perceived pressure from parents to ensure care was carried out in exactly the same manner as they did it at home.

*“When parents are such experts and they are watching you do everything, some people find that difficult or maybe the parents do it slightly different and might pick up on that and then people get a little bit worried.”* (participant 4)

However, because of the individualised care regimes of some children, there were limited opportunities to embed new learning in practice.

*“We don’t often get children with dialysis … it is almost like you need re-training each time … whereas if you are on intensive care or a dialysis ward it is just second nature isn’t it.”* (participant 35)

In general, participants valued the diversity of the children’s hospice role and welcomed the opportunity to provide holistic care and be involved with a range of tasks. Participants who had previously worked in NHS settings compared the two roles; the former being mainly focused on providing and documenting clinical care, with insufficient time to provide emotional support and little blurring of the traditional professional patient boundaries.

*“You have just got time, the quality time to spend with the patients and their families whereas in the hospital you have literally got ten minutes to see a patient regardless of whether they are upset or crying.”* (participant 30)

However, several participants found certain (and different) aspects of the role more challenging than others. All participants wanted to feel confident and competent, but the multiple and varied duties expected of them and the individualised and complex care needs of some of the children meant this was not always the case. Many participants identified on-going needs for training, mentoring and support to increase confidence in different aspects of the work.

### Team and organisational dynamics

The team was identified as the core unit of delivery and the contrast between a *‘team functioning’* well and one not was a central theme in the data. When asked, some participants were not able to explain what helped the team function well; others identified numerous factors that they believed could change how well individuals worked together as a team.

*“The team are very supportive of each other, but it can change depending on what’s going on … because the children might have changed, the team might have changed, sickness … sometimes the issues are very hard to get to the bottom of.”* (participant 4)

A poor functioning team was described as a key stressor. Conversely, when the team functioned well there was a calmer and happier atmosphere, harmony between colleagues and a sharing of efforts. Participants reported feeling more positive about work, found it easier to ‘switch off’ at the end of a shift and felt valued by colleagues, who were identified as the key source of informal support for the majority of participants.

*“For me, by far the biggest source of support is the informal support that comes from the other team members…just kind of in and amongst everything else.”* (participant 15)

Several participants emphasised the importance of the shared understanding and experience in the team, enabling staff to support each other.

*“Informally, I think the staff support we get is better than anywhere else I’ve worked because we are all doing something very similar and then we have got an understanding. If you have been dealing with a dying child other people have been there who are around you.”* (participant 10)

However, informal support was not always available. Working continuously alongside families restricted opportunities and some participants felt isolated from colleagues, particularly when they were providing intensive one to one care during a shift. Handover was identified as having some potential, but many participants observed that due to the amount of information to be transferred this rarely occurred. Handover was also, for some participants, a source of stress because the allocation of work, which occurred during handover, could lead to conflict between colleagues. This was linked to some participants not feeling confident in all aspects of the role, and worrying about being asked to take responsibility for certain tasks that they believed others in the team were more suited for.

Team conflict, which was identified as a key stressor by nearly all participants, was reported to impact negatively on team functioning and further isolate colleagues from each other. There were few opportunities to address conflict within the team and a perceived pressure to make sure families were not exposed to this. Some participants believed that being able to reflect constructively on work as a team helped to reduce conflict.

*“People feel bound together as a team because they are able to talk through things with each other, so it strengthens the team and they are able to work through and actually think of new approaches to a problem or issue or understand it better or maybe just learn from each other or how people tackle things.”* (participant 7)

For some participants, the organisation’s commitment to providing regular clinical reflection and other forms of support and training was seen as a way their own commitment to supporting children and families was acknowledged by the organisation. However although the organisation provided cover for staff to attend, many participants, including those working part-time or night shifts, were not able to attend as often as they would like.

Some participants who had in previous roles received clinical supervision, (a formal process of professional support and learning for individuals which enables them to develop knowledge and competence and help sustain wellbeing and quality work), were surprised this was not standard practice in the hospice and believed that the lack of clinical supervision contributed to a feeling of not being valued by the organisation.

*‘Being valued’* at work was important to participants and they welcomed feedback that their contribution was ‘making a difference’. This might come from the work itself, from families, or from their colleagues and the organisation.

*“We need to know that we are appreciated and it is all too easy not to say those things ... occasionally you know you will get a thank you … somebody would say ‘oh thanks it has been a really good shift’ and you would go out and you would feel nine foot tall you know.”* (participant 35)

Many participants welcomed the fact that whatever their background, they were treated as an equal member of the team because of the shared duties of care. However, sometimes this led to individual skills and experience being under-utilised. There was a feeling that the hospice could make greater use of the range of experience available.

*“There are so many people on the team who have got skills who could bring so much benefit to the team with regards to training, advice, education … but they don’t encourage people to use these skills.”* (participant 30)

Consulting and including the care team in developments within the organisation and drawing on their expertise was reported to help participants feel valued in their role. If changes that impacted on them happened without consultation, it caused anxieties about the organisation and their own role in it.

*“There are ways to implement changes and let people talk it out and see what there is, but you’re told to do it and that’s it you know, and it’s not always for the best… I just think if management listened more to staff. Because on the whole the staff here they’re damn good staff really.”* (participant 22)

Not being valued by the organisation was identified as a key stressor and one that could lead to feelings of resentment and a feeling of ‘us’ and ‘them’ between the team and senior management.

*“Sometimes we do get a pat on the back, but it’s not enough and I have said it before and I will say it openly again that the most important commodity… is the staff and that’s not what comes across.”* (participant 2)

The hospice itself, which was described as a peaceful and spacious setting with excellent facilities and equipment, was identified by many participants as an enabling factor and a rewarding aspect of work. Being able to park at work and enjoy home cooked food were also valued. Participants who had worked in NHS settings reported that the facilities and equipment were far superior in the hospice. However, some participants acknowledged that this could quickly be taken for granted.

### Individual resilience

All participants explained that factors outside of work could impact on their ability to cope with work-related challenges and experience the rewarding aspects of their work. Several participants described negative events in their own lives that had impacted on their resilience at work, and their capacity to provide emotional support to families and colleagues during these times.

*“When I had my own problems I struggled, well I struggled to come into work at all … I felt like I had lost confidence really and felt like very vulnerable with all the new machinery but that was partly my problem, what was going on in my life as well.”* (participant 35)

Participants described *‘individual coping’* strategies they used to help minimise the impact of work-related stressors on their lives, and the importance of creating some separation between work and home life. These included discussing particular events with a colleague at the end of a shift, using the drive home to reflect on work, or associating other practices with the ending of work, such as taking the dog for a walk or changing out of work clothes.

Only a small number of participants identified either formal or informal sources of support outside of work (e.g. external clinical supervision or close friends who worked in a similar role). The majority identified barriers to obtaining informal support outside work. These included patient confidentiality, the ‘taboo’ surrounding the work and not wanting to burden others who lacked the shared understanding of colleagues.

*“You would talk more with your colleagues about work related things but I would never go telling my friends … I just don’t think people understand what goes on in the hospice. I think it needs to stay at work.”* (participant 30)

Many participants expressed concerns about the wellbeing of colleagues they thought were at risk of burnout. Identifying one’s own vulnerabilities was perceived as more difficult, and a small number of participants who had experienced distress or burnout themselves had not seen the early signs.

*“You don’t always see clearly what your needs might be even if you’ve got the experience to draw on, like you know, I always find it easy to talk to people and share experiences; why wasn’t I doing it, you know?”* (participant 25)

Discussing individual vulnerabilities with colleagues or managers was identified as a challenge. Some participants felt an implicit pressure to be strong, like the families they supported.

*“If you are the type of person who you feel you should be coping, you won’t go and seek help because that means that you’re not, do you know what I mean? … You know I work at [children’s hospice], I work with death all the time so I should be able to manage it.”* (participant 9)

The limited support available outside work and the emotional intensity of the role, which many participants felt that only those working in this area would understand, created a greater need for formal provision from the organisation. Many but not all participants identified unmet needs for support. Several participants suggested that more training on work-related stress would help individuals identify their own strengths and vulnerabilities.

*“In caring situations lots of us are here because we need to be needed. I am a person who needs to be needed. I can’t help that. That is part of me but I know that and so I am aware of it … But some people don’t recognise it. That is the hard thing I think.”* (participant 1)

Whilst many conveyed passion about their work (describing what would traditionally have been called a vocational motivation), *‘job motivation’* varied across the sample and some participants believed that their needs as an employee (e.g., for professional development and training, maintaining a work-life balance) were sometimes overlooked because of the pressing priority of the children and their families. At the same time, participants valued the needs of children and their families being paramount. This conflict was evident in many accounts; wanting to get it right and make a difference, but at the same time wanting their own needs to be addressed.

*“It is about emotional investment, it is about commitment, it is about going the extra mile, and being alongside parents. But it’s sometimes more than that, that job can be done far, far better and far more effectively if there are systems in place in which people develop and grow.”* (participant 3)

## Discussion

In this study, three main, interlinking areas were found to impact on wellbeing at work: doing palliative care work; team dynamics and organisational factors; and individual resilience. Seven specific themes, contributing to these three main areas, identified work-related challenges and rewards, with the potential to both enhance and impede wellbeing at work. The study also sheds light on the interactive and synergistic relationship between these factors, and the impact that organisational structures and values, including the provision of support and development opportunities and the ethos of providing holistic care, can have on staff’s experience of working with children and their families in a hospice setting.

In line with other studies [13], the commitment of staff to deliver consistently high standards of clinical and holistic care in keeping with the children’s hospice philosophy was identified as a challenge, a reward and a motivator for the work. This might help to explain why individuals have high expectations of themselves, the team and the organisation, or conversely, how feelings of negativity and disappointment arise when they themselves, the team or organisational factors make this difficult to achieve, or when it is felt that the organisation does not value their commitment to providing quality care. The present study found that while the diversity in the hospice role was rewarding for staff, it could also cause anxieties when aspects of care were unfamiliar or at the limits of their own knowledge and skills. Providing regular training and support for the work is important to help staff feel confident and competent in what is increasingly recognised to be a multi-faceted and specialist role [11, 30].

The emotional investment in the work was acknowledged as rewarding and contributing to job satisfaction, but also sometimes placing individuals at risk of distress and potential burnout. McConnell et al., identified this as one of the key stressors for staff providing end-of-life care to children and young people [13]. Supporting work life balance, and ensuring regular opportunities to reflect on emotional and distressing aspects of work, is therefore not surprisingly, important to the staff. Enabling the team to function well was also important, helping individuals to ‘get it right’ and experience the rewards associated with this. Enabling individuals to support each other by addressing interpersonal conflict has been found to impact on quality of care and the rewards of work [23, 31].

The importance of individual factors and resilience emerged as significant in the present study, which revealed differences between participants in relation to their motivation to work in a children’s hospice, their relationship with the organisation itself, and their perceived risk of work-related stress and burnout. A ‘vocational motivation’ can contribute to resilience [32], as can recognition by the organisation for the work [33], including the provision of such mechanisms as training and appropriate clinical supervision [12]. Although there is some evidence that individuals can increase awareness of their own protective and vulnerability factors and so increase their own resilience [34], participants in the present study reported that it can be more difficult to recognise signs of stress and burnout in oneself than in others.

Support from colleagues and the organisation, with opportunities to discuss work in clinical reflection, were identified in this study as the primary resources for emotional support and learning. Other research also identifies these as important strategies [13, 31], partly because of the shared understanding among the staff about working with children and young people with life-limiting conditions, but also because of the need to explore and learn from events as a team. In this study, few participants reported being able to regularly and consistently attend structured group provision and some individuals expressed a need for more individualised clinical supervision. Traditionally, this has not been routinely provided to staff in palliative care settings [12], which is perhaps surprising considering the important role of clinical supervision for medical and nursing staff more generally [35].

Scales that measure work-related stressors and rewards have the potential to identify important factors affecting individuals [36], which could then be addressed through interventions like clinical supervision. However, while appropriate support and development opportunities are important, some of the stressors identified might be addressed through improved organisational practices, perhaps challenging existing notions that hospice environments do not suffer from the organisational difficulties present in other settings [13]. This was also highlighted in a recently published framework to support the wider hospice workforce [12], which found that “the quality of the work environment has a significant impact on stress levels, and most situations could be improved by effective leadership, a participatory culture and good line management of staff and volunteers” (p.5). The report recommends that organisations take the lead on developing and nurturing staff engagement. The present study suggests that in addition to providing appropriate support and training, involving staff in decision-making and change within the organisation and utilising the diversity of skills and expertise across the staff team can contribute to staff feeling more engaged.

## Conclusions

The specific challenges and rewards identified in the present study highlight some of the support and development that may be required for children’s hospice staff to feel competent and confident in what is a multi-faceted and emotionally demanding role. Limiting exposure to potential stressors and enhancing rewards through better support and development is a potentially useful strategy to help prevent work-related stress and burnout, which for participants in this study was perceived as a risk associated with the work of supporting children with life-limiting conditions and their families.

Organisational structures play a key role in ensuring that the emotional investment individuals make in delivering quality palliative care is valued, and that appropriate support, development and training for their role in supporting children and their families and their own wellbeing is available. Providing regular, structured and dedicated opportunities for staff to engage in clinical reflection or individualised supervision can provide a mechanism through which children’s hospice staff can be supported, and demonstrates an organisational commitment to staff wellbeing and development. Reducing barriers to discussing work-related stress and enhancing awareness about early signs of burnout are also important.
